# Compressibility of 304 Stainless Steel Powder Metallurgy Materials Reinforced with 304 Short Stainless Steel Fibers

**DOI:** 10.3390/ma9030161

**Published:** 2016-03-04

**Authors:** Bibo Yao, Zhaoyao Zhou, Liuyang Duan, Zhiyu Xiao

**Affiliations:** School of Mechanical and Automotive Engineering, South China University of Technology, Guangzhou 510640, China; yao.bibo@mail.scut.edu.cn (B.Y.); me201410100179@mail.scut.edu.cn (L.D.); zhyxiao@scut.edu.cn (Z.X.)

**Keywords:** powder metallurgy (P/M) material, compressive properties, fiber content, compaction pressure, high temperature nitriding

## Abstract

Powder metallurgy (P/M) technique is usually used for manufacturing porous metal materials. However, some P/M materials are limitedly used in engineering for their performance deficiency. A novel 304 stainless steel P/M material was produced by a solid-state sintering of 304 stainless steel powders and 304 short stainless steel fibers, which were alternately laid in layers according to mass ratio. In this paper, the compressive properties of the P/M materials were characterized by a series of uniaxial compression tests. The effects of fiber content, compaction pressure and high temperature nitriding on compressive properties were investigated. The results indicated that, without nitriding, the samples changed from cuboid to cydariform without damage in the process of compression. The compressive stress was enhanced with increasing fiber content ranging from 0 to 8 wt.%. For compaction pressure from 55 to 75 MPa, greater compaction pressure improved compressive stress. Moreover, high temperature nitriding was able to significantly improve the yield stress, but collapse failure eventually occurred.

## 1. Introduction

Porous metals have been widely investigated and applied due to their distinct permeability, sound and energy absorption properties, heat transfer and mechanical properties [1,2]. Nowadays, with the strict demands of different applications, many new types of porous metals with different geometries and microstructures are produced. The powder metallurgy (P/M) technique is a common method of producing porous metal materials. However, the applications of some P/M materials are limited in engineering for their performance deficiency. For example, sintered powder materials used as filters are too fragile to withstand heavy loads for liquid to pass through [3]. Therefore, the improvement of the mechanical properties is one of the key issues for the scientific and technological applications of porous metal materials.

Porous sintered stainless steel material has advantages in mechanical properties, corrosion and high temperature resistance. It mainly contains metal fiber sintered sheets (MFSSs) and P/M material. Many researchers have investigated its mechanical properties. Jin *et al.* [4] proposed a micromechanics random beam model about MFSSs to investigate the elastic-plastic behavior of the material. Zhao *et al.* [5] studied in-plane and transverse shear properties of MFSSs. Ducheyne *et al.* [6] investigated the tensile and compressive properties of porous austenitic stainless AIS 316L fiber material in experiment and theory. Kurgan [7,8] and Wang [9] revealed how the porosity, sintering temperature and environment influence the microstructure and mechanical properties of stainless steel P/M material, and built a model for the relationship between compressive strength and porosity.

Adding fiber is a method of improving the strength of metals. Fiber-reinforced metal matrix composites have high specific strength and modulus. To gain a large accessible strain interval to back-calculate fiber-reinforced materials accurately, Bucaille *et al.* [10] investigated the relationship between flow stress and penetration depth by nanoindentation tests. Isaacs [11], Bystricky [12] and Moseret [13] manufactured alumina fiber-reinforced aluminum matrix composites by pressure infiltration and investigated the mechanical properties. It was demonstrated that the fibers enhanced the yield stress of the metal. Okabe [14], Ochiai [15] and Jayalakshmi [16] revealed that the tensile strength of alumina fiber-reinforced aluminum matrix composites increased with increasing fiber volume fraction. Tungsten fibers 20 mm or less in diameter have been documented as improving the apparent work-hardening rate of pure copper [17]. The reference [18,19] noted that there was a strong non-uniformity of stress and strain between the matrix and fiber reinforcement, but the problem was not so obvious in fiber-reinforced P/M material. However, the study on improving the mechanical properties of P/M material by adding fibers is currently lacking in the literature.

Solution strengthening is also an appropriate hardening treatment method of improving the mechanical properties of porous stainless steel material. Hardness and strength of austenitic stainless steels can be enhanced by adding nitrogen [7,20]. Many references [21,22,23] have put forward a solution-nitriding mode to improve stainless steel strength, and have demonstrated the strengthening effect of high temperature nitriding. In addition to that, Bottoli [24] and Balusamy [25] have both indicated that nitriding treatment can improve the hardness of 304 stainless steels.

In summary, few reports have discussed the manufacture and mechanical properties of 304 stainless steel fiber-reinforced P/M materials, a new class of porous metal. In this study, the P/M material was produced via solid-state sintering of 304 stainless steel powders and 304 stainless steel fibers, which were alternately laid in layers according to mass ratio. Uniaxial compression tests were used to study the compression process of the P/M material. The effects of the fiber content, compaction pressure and high temperature nitriding on the compressive properties were investigated in detail.

## 2. Results and Discussion

### 2.1. Microstructural Characterization of the P/M Material

**Figure 1:**
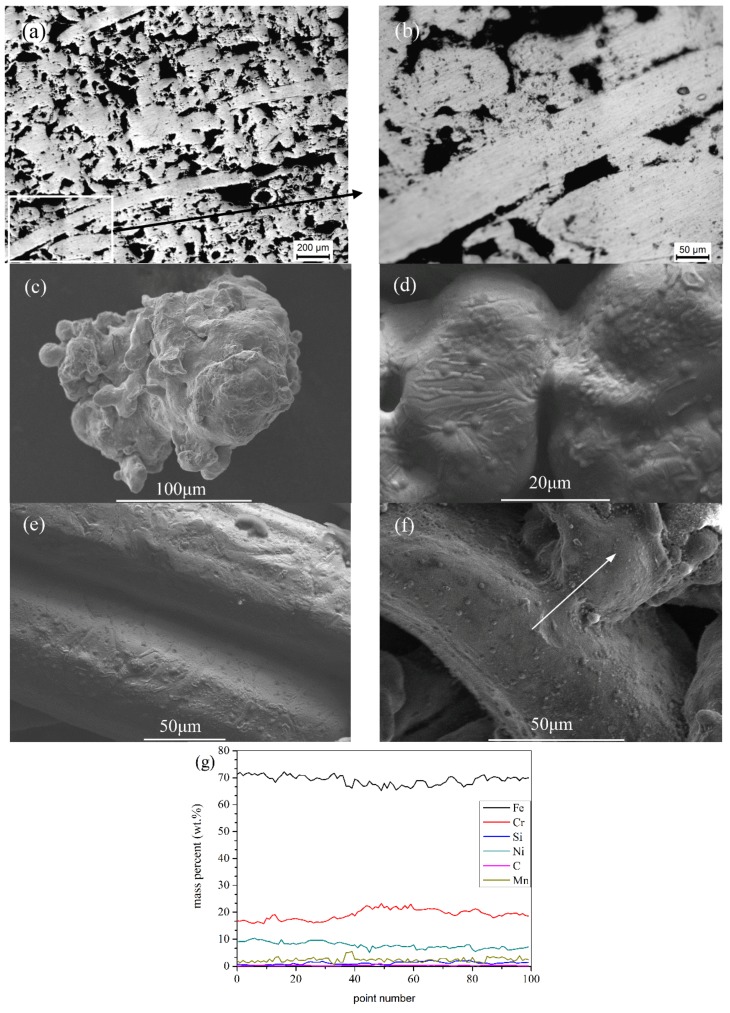
The metallographs of sample 2: (**a**) the structure of the P/M material in height direction; and (**b**) the area in high magnification; (**c**) irregularly shaped powder. SEM images of three sinteringjoints: (**d**) powder-to-powder sintering joint; (**e**) fiber-to-fiber sintering joint; and (**f**) powder-to-fiber sintering joint; (**g**) the linear EDS analysis of the sintering joint in (**f**).

In the manufacturing process before sintering, many contact regions are produced in green bodies. A number of sintering joints between powders and fibers, among powders, and among fibers are formed under the setting temperature and holding time as a result of material migration. Under high temperature, the amplitude of atomic vibration increases, which leads to its diffusion. More atoms in the contact area enter the range of atomic force so that the bonding surface forms. With the expansion of the bonding surface, the strength of the sintered body also increases. As the bonding surface is enlarged, the sintering neck is produced. The mass migration of atoms to the bonding surface causes the sintering neck to enlarge. Figure 1a,b show the structure in height direction (*i.e.* the vertical direction of layers) of sample 2. Many sintering joints can be found, reinforcing the strength of the P/M material. The average pore size is about 5.26 μm using the method of excluding gas and liquid. Many mountain-like microstructures of the irregularly shaped powders (Figure 1c) increase the contact area, which is beneficial to the formation of the sintering joints as a result of high surface energy, thereby enhancing the strength of the material. Further investigation reveals that there are three types of sintering joints presented in the material: powder-to-powder contact joints (Figure 1d), fiber-to-fiber joints (Figure 1e) and powder-to-fiber joints (Figure 1f). Figure 1g shows the liner EDS (energy-dispersive spectroscopy) analysis of the sintering joint in Figure 1f. The chemical composition is in accord with that of the fiber and powder, and the element contents have few fluctuations. It has been confirmed that material migration happens thoroughly in the formation of sintering joints. As the samples are cuboids, the density can be calculated by the quality-volume method. The P/M samples and their parameters are given in Table 1.It should be noted that the material has higher porosity, ranging from 47.50% to 60%, while the porosity of normal P/M material is about 5%–15% [26].

### 2.2. Compressive Process of the P/M Material

**Figure 2:**
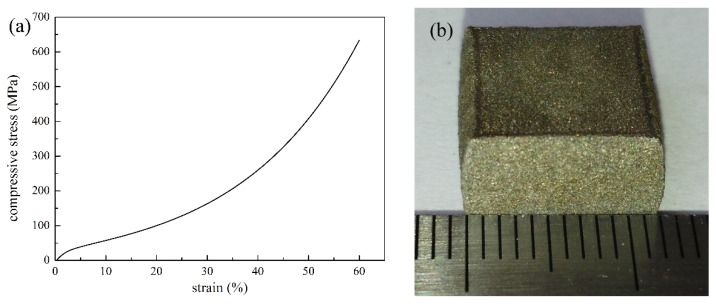
(**a**) Compressive curve; and (**b**) the picture after compression test of sample 2.

The compressive process of highly porous stainless steel P/M materials and sintered metal fiber sheets contains three stages: the linear-elastic stage, the plastic deformation stage, and the densification stage [27,28]. The stress-strain curve of sample 2 is shown in Figure 2a, which is different from the description in the literature [27,28]. At the beginning, the P/M material experienced a short-term elastic stage, up to 1% of strain, where stress increased linearly with increasing strain. Partially reversible deformation of fibers and powders occurred. Then, the stress slowly increased with large strain in the following plastic deformation stage. With the increase of strain up to 35%, almost all powders and fibers plastically deformed. At this period, the pores became smaller and smaller, and the shape of the pores changed irrecoverably. The propagation of the force inside the P/M material was blocked due to the existence of pores in the compression process, so the compressive stress increased slowly with increasing strain. After entering the densification stage, stress increased sharply with increasing strain. Meanwhile, the sample changed from cuboid to cydariform (Figure 2b) without collapse in the compression process when the force reaches the max value, 70 kN.

### 2.3. Effect of Fiber Content on the Compressive Properties of the P/M Material

Fiber content is one of the important parameters of the P/M material and has a significant impact on the compressive properties of samples.

**Figure 3:**
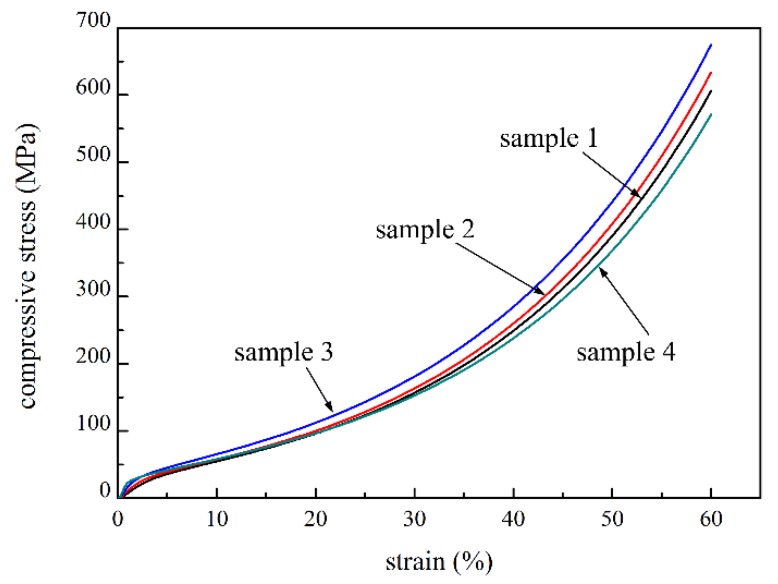
Compressive curves of the compacted sample 1–4 with 4 wt.%, 6 wt.%, 8 wt.% and 0 wt.% fiber content, respectively.

**Figure 4:**
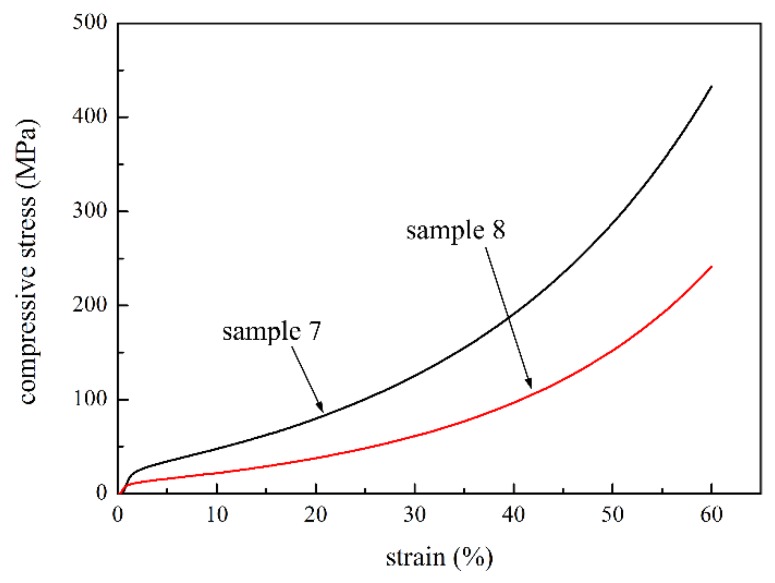
Compressive curves of the loosely sintered sample 7 and 8 with 6 wt.% and 0 wt.% fiber content, respectively.

To study the effect of fiber content on the compressive strength, the compacted P/M materials with four fiber contents of 4 wt.%, 6 wt.%, 8 wt.% and 0 wt.% (sample 1, 2, 3 and 4, respectively) were selected to carry out compression tests. The compressive curves of the materials with different fiber contents are shown in Figure 3. For all of the samples, the compressive process experienced three stages, similar to those of sample 2. Figure 3 reveals that the compressive stress is clearly improved in the densification stage with increasing fiber content in the same strain, and the differences were greater with increasing strain. The strength and elasticity modulus of fiber were higher than those of powder, so fiber deformed less from the same stress. In addition, the increasing of fiber content was able to reduce the micro-hole in the sectional area, so as to decrease the stress concentration. The average pore size of sample 4 was 5.75 μm, while that of sample 2 was 5.26 μm. The pore size reduced with the increasing fiber content, and the flow stress increased with a decrease in pore size [29]. Furthermore, the P/M material with higher fiber content had lower porosity, increasing the effective bearing and contact area, thus forming more sintering joints in the sintering process [30]. These worked to increase the compressive strength of the P/M material with higher fiber content. The compressive properties of the loosely sintered P/M materials (sample 7 and 8) were also investigated, as shown in Figure 4. It is found that the change rules of stress-strain curves are similar to those of the compacted samples. Compressive stress improved greatly with increasing fiber content.

### 2.4. Effect of Compaction Pressureon the Compressive Properties of the P/M Material

**Figure 5:**
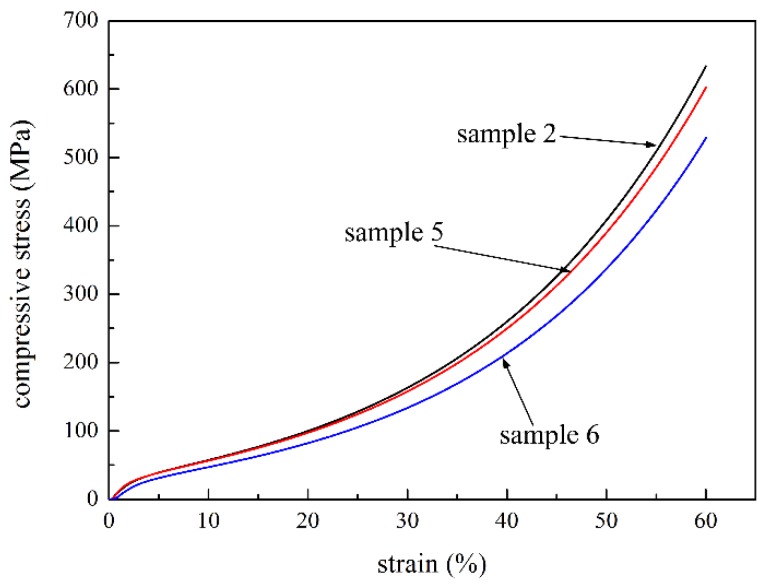
Compressive curves of samples 2, 5, and 6 with 75 MPa, 65 MPa and 55 MPa pressure, respectively.

Compaction pressure has a great influence on the formation of green bodies, and eventually affects the compressive strength. Samples 2, 5 and 6 were formed under the pressure of 75 MPa, 65 MPa and 55 MPa, respectively, and their compressive properties were compared. The compressive stress-strain curves of all the samples had similar trends. Figure 5 shows that the compressive stress almost increased with the increase of compaction pressure under the same strain. The results are attributed to the following reasons: Fibers bent and powders deformed under compaction pressure, thus increasing the contact area; larger compaction pressure led to lower porosity and a smaller pore size (e.g., the average pore sizes of sample 2 and 5 are 5.26 μm and 9.56 μm, respectively); thus, more fibers, powders and sintering joints distributed in the cross section could be used to withstand the compressive load. Therefore, larger compaction pressure could improve the compressive strength of the P/M material.

### 2.5. Effect of High Temperature Nitriding Heat Treatment on the Compressive Properties of the P/M Material

High temperature nitriding heat treatment can affect the compressive properties of stainless steel materials [21,22,23]. The compressive strength of the P/M material can be greatly improved by nitriding.

**Figure 6:**
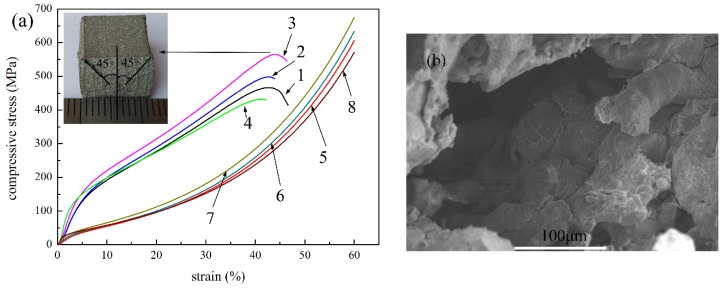
(**a**) Compressive curves of the compacted samples: after nitriding: sample 1 (curve 1); sample 2 (curve 2); sample 3 (curve 3); sample 4 (curve 4); before nitriding: sample 1 (curve 5); sample 2 (curve 6); sample 3 (curve 7); sample 4 (curve 8); (**b**) the cracking character picture of the nitrided sample 2 after compression test.

Figure 6a shows the experimental compressive stress-strain curves of the compacted P/M materials (sample 1, 2, 3 and 4) with and without nitriding treatment. The nitrided P/M materials experienced a short-term elastic deformation stage, followed by a plastic deformation stage with small increases of compressive stress to large strain. On reaching the maximum value, the stress began to decrease slowly with increasing strain until the collapse occurred. The compressive strength is defined as the peak stress. A crack began to show in the weak middle area of the samples. As the compression continued, the sintering joints, fibers and powders began to irreversibly deform and fracture, quickly causing the destruction of the material. During the fracture propagation, the stress increased, and there was a corresponding increase in the size of the damage zone, which promoted the crack nucleation or deflection [31]. Eventually, the fracture occurred at a 45° angle relative to the compressive loading direction, which was in the direction of maximum shear stress. The cracking character is shown in Figure 6b. The compression process was different from that of the untreated sample, not experiencing the densification stage. The results are consistent with the maximum shear stress criterion of the plastic flow failure mode of metal materials. The phenomenon can be explained by the following reasons: Active nitrogen atoms spread from the surface to the core, and dissolved in the stainless steel atoms, giving rise to lattice deflection; stress fields in steel grains prevented dislocation movement, which is the main reason of plastic deformation of metals; thus, the strength of powders, fibers, and sintering joints were greatly improved while the plasticity reduced. Therefore, compressive stress was improved greatly and the fracture occurred when the shear stress reached its ultimate value.

As seen in Figure 6a, compressive strength was obtained when the strain wasabout 40%, and the strength of samples 1, 2, 3 and 4 were about 466.12 MPa, 497.74 MPa, 565.22 MPa and 431.75 MPa, respectively, while the compressive stresses were 249 MPa, 259.88 MPa, 284.83 MPa, 237.81 MPa, respectively, in 40% strain without nitriding. It is clear that the elastic deformation stages were longer than those of untreated samples, and the yield stress significantly improved with nitriding. The porous materials applied in the mold industry usually stand compression. Therefore, the improvement of yield strength could enlarge the application of porous materials. Finally, the stress decreased as the crack came into being until the samples were completely damaged for their poor plasticity.

**Figure 7:**
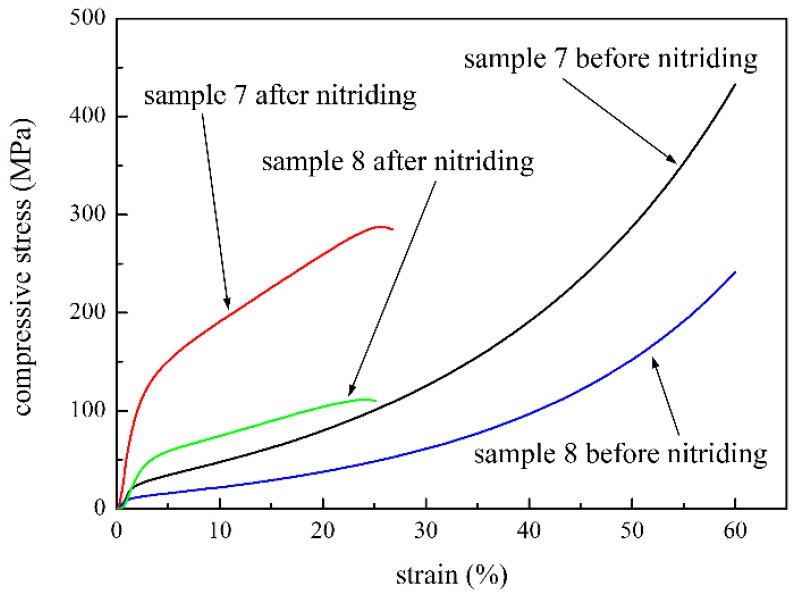
Compressive curves of the samples with and without nitriding by loose sintering.

Figure 7 shows the compressive curves of the loosely sintered sample 7 and 8 with and without nitriding. The change trend of compressive curve is similar to that of the compacted sample. The elastic deformation stage is also enlarged, and the stress is greatly improved with nitriding. Under the 25% strain, the strength of samples 7 and 8 with nitriding are 286.78 MPa and 110.41 MPa and those without nitriding are 100.47 MPa and 48.31 MPa, respectively. It should be noted that the nitriding heat treatment had less effect on the compressive properties of the loosely sintered samples. The compacted specimen had more contact points between powders and fibers, and among powders; therefore, compared to loosely sintered specimens, coarser sintering joints were produced in the sintering process. The strength of sintering joints showed greater improvement; thus, the compressive strength of the compacted P/M material increased more than that of the loosely sintered samples.

## 3. Experimental Procedures

### 3.1. Manufacturing Process of the P/M Material

**Figure 8:**
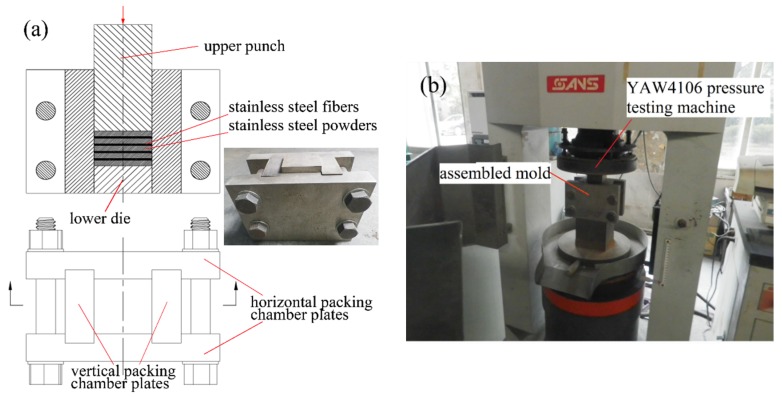
(**a**) The photograph of assembled mold, alternatepowder and fiber layers; (**b**) compaction process.

The manufacturing processing procedure of the P/M material was divided into the following five steps: fiber chipping, green bodies manufacturing, sintering, heat treating and testing. First, the short 304 stainless steel fibers with length from 5 to 8 mm were fabricated by cutting 304 stainless steel wires (0.8 mm in external diameter, 100 μm in fiber diameter. Foshan Nanhai Liangjunyin Stainless Steel Products Co., Ltd., Foshan, China) with a short-cutting machine. Next, the materials with a mixture of 304 stainless steel powders(Haining Feida Metallurgical Powder Co. Ltd., Haining, China) and fibers were put into the packing chamber of aspecially assembled mold by alternately laying powders and fibers manually in layers (as shown in Figure 8a) according to the mass ratio. Then, the compaction pressure was applied on the upper punch for half a minute to produce green bodies. The pressure was controlled by the computer controlled servo pressure testing machine (YAW4106, Shanghai Sans Testing Machine Co. Ltd., Shanghai, China), as shown in Figure 8b. Subsequently, the green bodies were put in a sintering box for sintering. The sintering was carried out in the pressurized air cooling vacuum sintering furnace (WHS-20, Zhongshan Kaixuan Vacuum Technology and Engineering Co. Ltd., Zhongshan, China), providing a vacuum atmosphere with a vacuum degree of about 5 × 10^−2^ Pa. A stage heating method was used to optimize the heating rate. The heating rate was kept at 10 °C·h^−1^ when the temperature was below 800 °C, and was reduced to 5 °C·h^−1^ as the temperature rose above 800 °C. Then, the temperature was kept in 1350 °C for 60 min. Finally, the samples were cooled in the furnace to room temperature. Compared to fiber-reinforced P/M materials, the materials without fibers were produced by the same process as described above, but without laying fibers. The loosely sintered materials were produced by the same laying method without compaction pressure, and then sintered as described above.

Nitriding treatment was conducted on samples in the modified vacuum sintering furnace. The furnace was vacuumized at first. Then, the stage heating method was adopted for the heat period. When the temperature was below 800 °C, the heating rate was kept at 10 °C·h^−1^, and the heating rate was decreased to 5 °C·h^−1^ with the temperature above 800 °C. When heating temperature reached 1200 °C, the evacuation system was closed and nitrogen gas was then filled until the pressure reached 0.12 MPa. This temperature and pressure were held for about 240 min. Finally, the samples were cooled in the furnace to room temperature.

### 3.2. Characterizations and Compression Test of the P/M Material

Since the sample has a regular geometric shape, the average porosity can be calculated using the quality-volume method by the following equation according to the reference [3]:

(1) $$\varepsilon =(1-\frac{m}{\rho_{s}V})\times 100\%$$

where *ε* is the porosity of the P/M material; *ρ*_s_ is the density of 304 stainless steel (g/cm^3^); *m* is the mass of the P/M material (g); and *V* is the volume of the P/M material (cm^3^).

The pore size was measured by an aperture analyzer (PSDA-20, Nanjing Zhongchao Environmental Protection Science and Technology Co., Ltd., Nanjing, China) via excluding gas and liquid. The samples were 30mm in diameter, and 3mm in thickness. At first, the samples were put in absolute ethanol for an hour. Then, the samples were fixed in an aperture analyzer. Nitrogen was blown, and the pressure was controlled to dry the samples gradually.

The microscopic structure of the P/M material was observed by a scanning electron microscope (S-3700N, Tokyo, Japan) equipped with energy-dispersive spectroscopy (EDS) and an inverted metallurgic microscope (DMI 5000M, Germany). The compression test was carried out on a universal material testing machine (AG-100NX, Shimadzu, Kyoto, Japan) with a capacity of 100 KN. Each test analyzed three samples, and the average was the final result. The maximum compression force was applied at about 70 KN in the experiment. The sample was 15 mm in length, 10 mm in width, and 10 mm in thickness. All samples were compressed at a constant load speed of 1 mm/min. The data collection system of the machine was used for data acquisition, and the data were saved every 0.01 s. All tests were conducted at ambient temperature (approximately 25 °C).

## 4. Conclusions

A P/M material was produced via the solid-state sintering of stainless steel powders and fibers by alternately laying powders and fibers manually in layers according to mass ratio. An investigation on its compressive properties provided the following key findings: 

(1) The compression process of the P/M material without nitriding includes the following three steps: linear-elastic stage, plastic deformation stage, and densification stage. The compression process firstly experienced a short-term elastic stage and then entered a yield stage with no obvious platform; finally, the stress increased steeply, with the strain increasing in densification stage. During the process, the sample changed from cuboid to cydariform without damage. After nitriding, the densification stage did not present, and the strength significantly improved, but the entire structure was eventually destroyed. In addition, the angle of fracture direction was approximately 45° relative to the compressive load direction.

(2) Fiber content, compaction pressure, and high temperature nitriding heat treatment had significant effects on the uniaxial compressive properties of the P/M material. The compressive stress increased with increasing fiber content and compaction pressure under the same strain. Compared to fiber content and compaction pressure, nitriding heat treatment had the greatest influence on compressive properties. The compressive strength greatly improved, and the elastic deformation stage was enlarged with nitriding. Nitriding affected the compressive strength of the compacted P/M material more than that of the loosely sintered samples.

## Figures and Tables

**Table 1:** The P/M samples and their parameters.

Sample Number	Powder Particle Size (mesh)	Fiber Diameter (μm)	Fiber Content (wt.%)	Compaction Pressure (MPa)	Density (g/cm^3^)	Porosity (%)
1	100	100	4	75	4.16	47.5
2	100	100	6	75	4.19	47.2
3	100	100	8	75	4.23	46.7
4	100	100	0	75	4.15	47.7
5	100	100	6	65	4.13	47.9
6	100	100	6	55	3.99	49.7
7	200	100	6	0	3.81	52.0
8	200	100	0	0	3.17	60.0
